# Long-Term Survival and Its Related Factors in Pediatric Liver Transplant Recipients of Shiraz Transplant Center, Shiraz, Iran in 2012

**DOI:** 10.5812/hepatmon.10257

**Published:** 2013-07-08

**Authors:** Najmeh Haseli, Jafar Hassanzadeh, Seyed Mohsen Dehghani, Ali Bahador, Seyed Ali Malek Hosseini

**Affiliations:** 1 Department of Epidemiology, School of Health and Nutrition, Shiraz University of Medical Sciences, Shiraz, IR Iran; 2 Department of Pediatric Gastroenterology, Organ Transplantation Center, Namazi Teaching Hospital, Shiraz University of Medical Sciences, Shiraz, IR Iran

**Keywords:** Survival, Pediatrics, Liver Transplantation, Iran

## Abstract

**Background:**

Liver transplantation is considered as the standard treatment for both children and adults with end-stage liver diseases. Using this method, children who have no chance for life can live a much longer life .Shiraz Transplant Center is the major pediatric liver transplant center in Iran. Therefore, determining patients’ survival and its effective factors can help clinical programming for increasing such patients’ survival after liver transplantation.

**Objectives:**

The present study aimed to investigate the survival of patients below-18-years-old undergoing liver transplantation and the factors affecting their survival.

**Patients and Methods:**

The present historical cohort study was conducted on 392 patients below-18-year-sold who had undergone liver transplantation for the first time in the Namazi hospital liver transplant center, Shiraz, Iran between 2000 and 2011. In this study, 1-, 3-, 5-, and 10-year survival of the patients was assessed using Kaplan-Meier and life table methods. The effect of factors related to the recipients, donors, and the transplantation process on the patients’ survival was also investigated.

**Results:**

*According* to the results, 1, 3, 5 and 10-year survival of patients was 73%, 67%, 66%, and 66%, respectively. Besides, 1 ,3, 5, and 10-year survival of the patients who survived 1 and 3 months after the transplantation was 84%, 78%, 77%, and 77% and 89%, 82%, 81%, and 81%, respectively. In the univariate analysis, age, patients’ weight at transplantation, initial diagnosis, PELD/MELD score, existence of post-transplant complications, and year of transplantation were found to be effective factors on the patients’ survival. In the multivariate analysis, only the type of graft, PELD/MELD score, and existence of post-transplant complications were the prognostic variables.

**Conclusions:**

In this study, the patients’ survival rate was 73%, which is quite low compared to the survival rate reported in other studies. Although we only have a 12-year experience with pediatric liver transplantation, the survival rate has increased in our center through the recent years (2008-2011). However, the survival rate of the patients who had survived 3 months after the transplantation was 89% which is comparable to other studies. Overall, cholestatic diseases (biliary atresia was the most prevalent), type of transplantation (split), PELD/MELD score > 20, and existence of post-transplant complications increased the risk of death after the transplantation.

## 1. Background

Liver transplantation is the standard treatment for patients with end-stage liver diseases and is accompanied by high success rate in patients who cannot be treated by any other method. Nowadays, liver transplantation is routinely performed around the world. Moreover, its success rate, which is assessed as 1-year survival, has increased from 30% in the 1970s to 90% today (1-5). Due to this success rate, the number of liver transplantations is increasing every year. However, demand for liver transplantation is much more than what is available; in a way that more than 17000 patients were in the waiting list for liver transplantation in June 2012 in the U.S. (6). Overall, children comprise 15-20% of patients in liver transplantation waiting lists. However, because children below 5 years old have the highest mortality rate in comparison with other age groups and liver transplantation is the only acceptable standard treatment, which can save them from death, this group of patients are more important (7). The first liver transplantation was done by Dr. Starzl on a 3-year-old child in 1963 which finally resulted in death (1-6). In Iran, the first liver transplant on a child was performed in Namazi hospital, Shiraz in 1999 (8). In general, indications for liver transplantation in children include cholestatic diseases, metabolic disorders, acute liver failure resulting from viral infections or drug consumption, chronic hepatitis, cirrhosis, and malignancies (4). Nevertheless, biliary atresia is the most prevalent disease, which leads to liver transplantation in children. Although, performing surgery during infancy improves the symptoms of the disease to some extent, 75% of such children will need liver transplantation before the age of 5 because of recurrent cholangitis and biliary cirrhosis (9). Goss et al. reported the 5-year survival of these patients after the transplantation as 78% (10). Liver transplantation also plays a major role in congenital metabolic disorders; however, it is only recommended in cases when the disease is only in the liver and is treated by transplantation, such as in Crigler-Najjar syndrome, or when the extra-hepatic enzyme abnormality is repaired, such as in tyrosinemia. Moreover, the extra-hepatic symptoms of the metabolic disorder, such as the nervous complications of Wilson’s disease, should not impede the transplantation (11). In various studies, 1 and 5-year survival rates have been reported as 90% and 85%, respectively (12-14). Determining the best time for liver transplantation as well as the factors affecting both short-term and long-term survival of liver transplant patients are considered as debatable issues in this field (15). Although more than a decade has passed from performance of pediatric liver transplantation in Iran, no studies have been conducted on the children’s survival rate and its effective factors after transplantation. In addition, this surgery is one of the most expensive interventions for both the patients and the health system.

## 2. Objectives

The present study aims to investigate the survival of patients below-18-years-old undergoing liver transplantation and the factors affecting their survival.

## 3. Patients and Methods

The present study was a survival analysis which was performed in the form of a historical cohort study. The study population included all of the 392 patients below-18-years-old who had undergone liver transplantation for the first time in Namazi hospital, Shiraz, Iran between April 2000 and March 2011. The patients’ information which was collected from their records included; the recipients’ characteristics, such as age, sex, weight at transplantation, blood group, initial diagnosis of liver disease, CHILD score, and PELD/MELD score; donors’ characteristics, including age, sex, and blood group; and the transplant features, including the type of graft, surgical technique, year of transplant, existence of post-transplant complications, and length of hospital stay after the transplantation. In addition, the information about the patients’ follow up was completed through a phone conversation with the patients.

### 3.1. Ethics Approval

The present study was approved by the Ethics Committee of Shiraz University of Medical Sciences, Shiraz, Iran. Inaddition, informed consents for obtaining the information as well as the follow-up data were taken from all the participants or their families.

### 3.2. Statistical Analysis

The data were analyzed using the Kaplan-Meier and life table non-parametric methods as well as Cox regression model. In Kaplan-Meier and life table methods, survival was computed based on different variables in a univariate manner and compared between different categories of the variables using the Log-Rank and Generalized willcoxon (Breslow) tests. Next, the significant variables of Kaplan-Meier and life table methods and those with P < 0.2 were entered into the Cox regression model .Proportional hazard assumption was assessed by drawing Log[-log(t)] on Log (t) Figures. All the statistical analysis was performed using the SPSS statistical software (v.16). It should be mentioned that the starting point of the patients’ survival analysis was the time of the liver transplantation, while the end point was considered as the patients’ death or retransplantation.

## 4. Results

Among the 392 transplanted patients, 229 (58.4%) were boys, while 163 patients (41.6%) were girls. The mean ± SD of the patients' age and weight at transplant was 8.5 ± 5.7 years and 26.3 ± 17.5 kg, respectively. Moreover, 4.9%, 63.5%, and 31.6 % of the patients were below 1, between 1 and 12, and between 12 and 18 years old, respectively. In addition, blood group of 36.3%, 23.3%, 6.5%, and 33.9% of the patients was A, B, AB, and O, respectively. Categorization of the patients based on the CHILD scoring system showed that 22.6%, 48.4%, and 29% of the patients were in class A, B, and C, respectively. In this study, 38 %of the cases had congenital metabolic disorders, 33% had cholestatic diseases, 11.9% had hepatitis, 1% suffered from malignancies, and 15.8% had other causes (Table 1). The mean ± SD of PELD/MELD scores was 20.3 ± 8.9 and 44.1% of the patients had PELD/MELD scores < 20. Considering the donors, 53.4% were female and 46.6% were male. The mean age of the donors was 25.7 ± 10.8 years. In addition, the blood group of 27.5%, 26%, 4.5%, and 42% of the donors was A, B, AB, and O, respectively. Considering the surgical technique, 32.7%, 3.2%, 27%, 27.4%, and 9.7% were piggyback, standard, left lobe, left lateral segment, and right lobe, respectively. Overall, 38.8%, 49.4%, and 11.9% of the patients received whole, partial living related, and split transplants, respectively. Moreover, 58.4% of the patients showed at least one early or late complication (such as acute cellular rejection, infection complication, biliary complication, hepatic artery thrombosis, cardiopulmonary problems, renal problems and so on) after the transplantation, while no complications were found in 41.6% of the patients. The mean ± SD of post-transplant hospitalization and follow up time were 17.1 ± 13 days and 25.2 ± 27.8 months, respectively. Regarding the year of transplant, 6.4%, 23.4%, and 70.2% of the transplantations were done between 2000-2003, 2004-2007, and 2008-2011, respectively.

**Table 1.** Etiology of Liver Transplantation in the Below-18-Year-Old Patients Transplanted in Namazi Hospital Liver Transplant Center, Shiraz, Iran

Etiology of Liver Transplant	No. (%)
**Congenital Metabolic Disorder**	
Wilson’s Disease	56 (14.3)
Tyrosinaemia	43 (11)
Crigler Najar(type1)	30 (7.7)
Familial Hypercholesterolemia	19 (4.8)
α1-anti Trypsin Deficiency	1 (0.2)
Total	149 (38)
**Cholestatic Disease**	
Biliary Atresia	56 (14.3)
PFIC^a^	47 (12)
PSC	10 (2.6)
Neonatal Hepatitis	10 (2.6)
Caroli Disease	5 (1.3)
Intrahepatic Bile Duct Paucity	2 (0.5)
Total	130 (33.3)
**Hepatitis**	
Autoimmune Hepatitis	47 (10.7)
Hepatitis B	4 (1)
Hepatitis C	1 (0.2)
Total	47 (11.9)
**Malignancy,Hepatocellular Carcinoma**	4 (1)
**Other**	
Cryptogenic cirrhosis	56 (14.3)
Fibro Polycystic Liver Disease	4 (1)
Budd-Chiari Syndrome	2 (0.5)
Total	62 (15.8)
**Total**	392 (100)

^a^ Abbreviations: PSC, Primary sclerosing cholangitis; PFIC, Progressive familial intrahepatic cholestasis

Among the 392 patients under study, 274 (69.9%) survived and 118 patients (30.1%) died after the transplant because of early or late complications and rejection; however, the time of death was known for only 108 cases. It should be noted that 50 (46.3%) and 78 (63%) of the deaths occurred in the first month and the first trimester after the transplantation, respectively. However, no deaths occurred among the patients who survived 5 years after the transplant. The survival rates of all the patients and those who survived at least 1 and 3 months after the transplantation are presented in Table 2. As the Table depicts, after excluding the patients who had died in the first and the third month post-transplantation, 1-year survival was obtained as 84% and 89%, respectively.

**Table 2.** Estimation of the 10-Year Survival of Patients Below-18-Years-Old Transplanted in Namazi Hospital Liver Transplant Center Who Had Survived at Least 1 and 3 Months After the Transplantation Using the Kaplan-Meier Method

Category	Cumulative Survival, %						
	**1-Month**	**3-Months**	**6-Months**	**1-Year**	**3-Years**	**5-years**	**10-Years**
**All patients**	86	82	78	73	67	66	66
**Patients Who Survived at Least 1 Month After the Transplantation**	96	92	89	84	78	77	77
**Patients Who Survived at Least 3 Months after the Transplantation**	96	95	92	89	82	81	81

**Figure 1.**
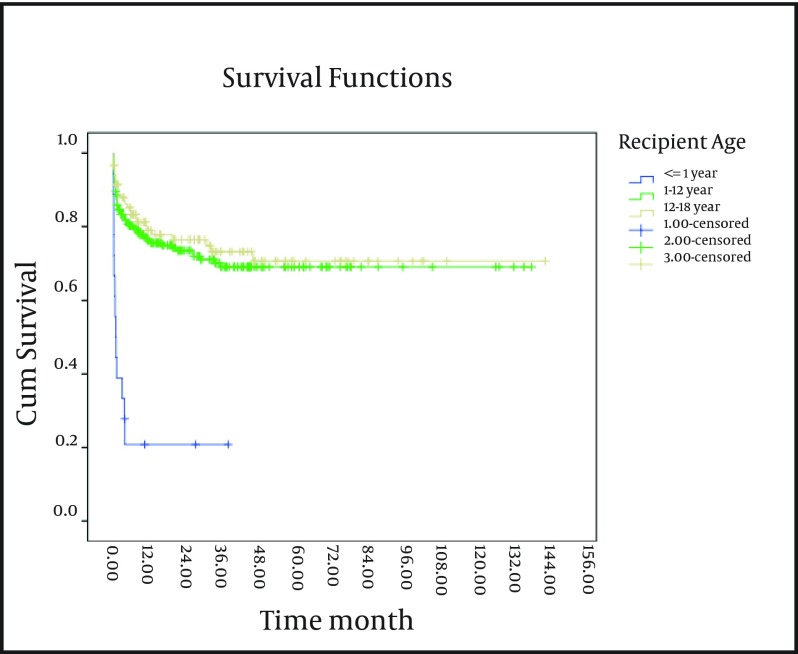
The 10-Year Survival Curve of the Patients Below-18-Years-Old Transplanted in Namazi Hospital Liver Transplant Center Based on Age Group

In the present study, the survival rate of patients, below-one-year-old, was lower than the other age groups (P < 0.001) (Figure 1).

Nevertheless, no significant difference was observed between the patients’ survival rates based on the recipient’s sex, blood group, and CHILD category, donor’s age, sex, and blood group, surgical technique, and length of hospital stay after the transplantation. 

The patients’ survival rate based on the initial diagnosis of the liver disease and the patient’s weight at transplantation using the life table method is presented in Tables 3 and 4.

**Table 3.** Estimation of the Survival Rate of the Patients Below-18-Years-Old Transplanted in Namazi Hospital Liver Transplant Center Based on the Initial Diagnosis Using the Life Table Method

	Metabolic Disease		Cholestatic Disease		Hepatitis Disease		Malignancy Disease		Other Disease	
	SR^b^, %	SE^a^	SR^b^, %	SE^a^	SR^b^, %	SE^a^	SR^b^, %	SE^a^	SR^b^, %	SE^a^
**1 year**	77	0.13	61	0.07	78	0.23	56	0.29	81	0.23
**2 years**	76	0.77	60	0.59	78	0.32	56	0.41	74	0.46
**3 years**	69	0.77	57	0.69	74	0.79	56	0.51	74	0.62
**4 years**	67	0.99	57	0.79	74	1.07	56	0.59	74	0.74
**5 years**	67	1.19	57	0.88	74	1.3	-	-	74	0.84
**6 years**	67	1.36	57	0.97	74	1.49	-	-	74	0.94

^a^Standard error of survival rate

^b^Survival rate

**Table 4.** Estimation of the Survival Rate of Patients Below-18-Years-Old Transplanted in Namazi Hospital Liver Transplant Center Based on the Recipient’s Weight Using the Life Table Method

	Weight, > 1 SD Above Mean		Weight, 0-1 SD Above Mean		Weight, 0-1 SD Below Mean		Weight, > 1 SD Below Mean	
	SR^b^, %	SE^a^	SR^b^, %	SE^a^	SR^b^, %	SE^a^	SR^b^, %	SE^a^
**1, year**	78	0.22	79	0.20	68	0.58	29	0.04
**2, years**	78	0.31	73	0.53	65	0.38	29	0.06
**3, years**	75	0.79	69	0.84	61	0.49	14	0.08
**4, years**	68	0.97	69	1.08	61	0.60	14	0.1
**5, years**	68	1.17	69	1.27	61	0.69	-	-
**6, years**	68	1.34	69	1.44	61	0.77	-	-

^a^Standard error of survival rate

^b^Survival Rate

**Figure 2.**
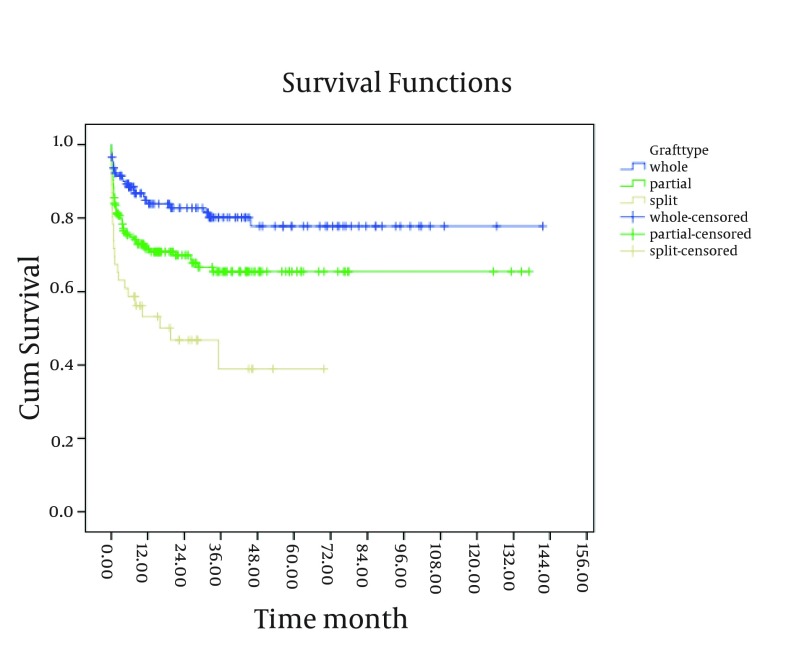
The 10-Year Survival Curve of Patients Below-18-Years-Old Transplanted in the Namazi Hospital Liver Transplant Center Based on the Graft Type

A significant relationship was observed between the patients’ survival rate and the cause of transplantation; in a way that the patients with malignancy and cholestatic disorder had lower survival rates compared to the others (P < 0.05). Moreover, the survival rate of the patients with weight > 1 SD below mean, at transplant was lower than that of the other groups (P < 0.001). The study findings revealed a significant relationship between the patients’ survival rate and the graft type (Figure 2). As shown in Figure 2, the patients who received a split had a lower survival rate compared to the others (P < 0.05). 

**Figure 3.**
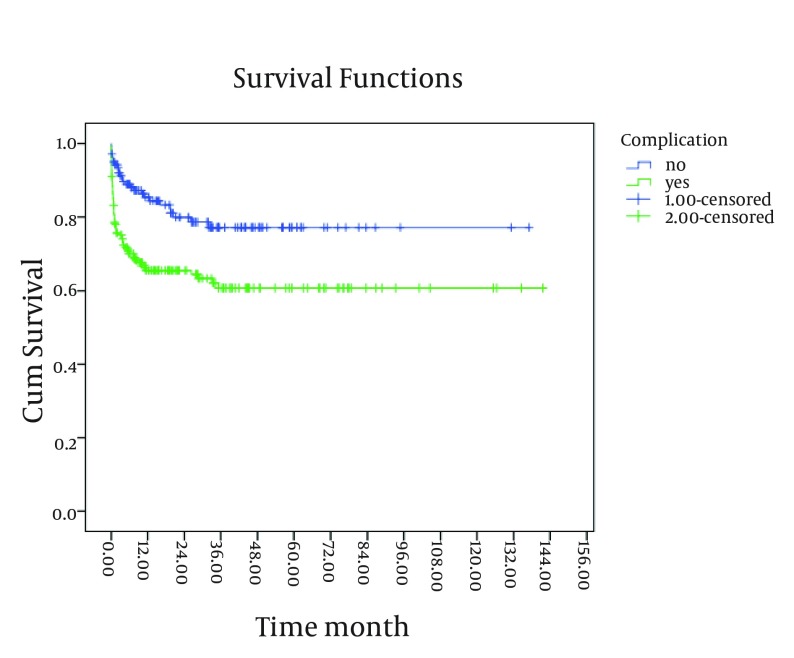
The 10-Year Survival Curve of Patients Below-18-Years-Old Transplanted in the Namazi Hospital Liver Transplant Center Based on the Existence of Post-Transplant Complications

**Figure 4.**
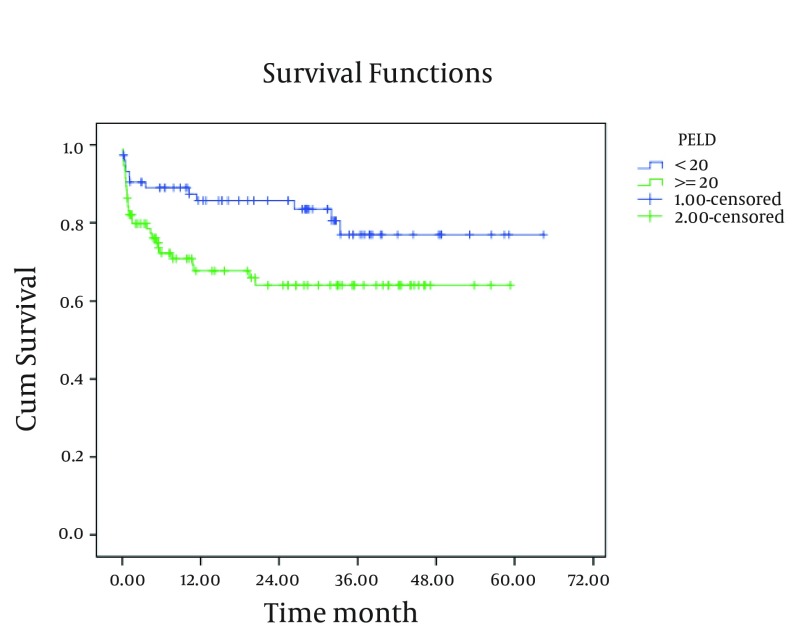
The 10-Year Survival Curve of Patients Below-18-Years-Old Transplanted in Namazi Hospital Liver Transplant Center Based on PELD / MELD Score

**Figure 5.**
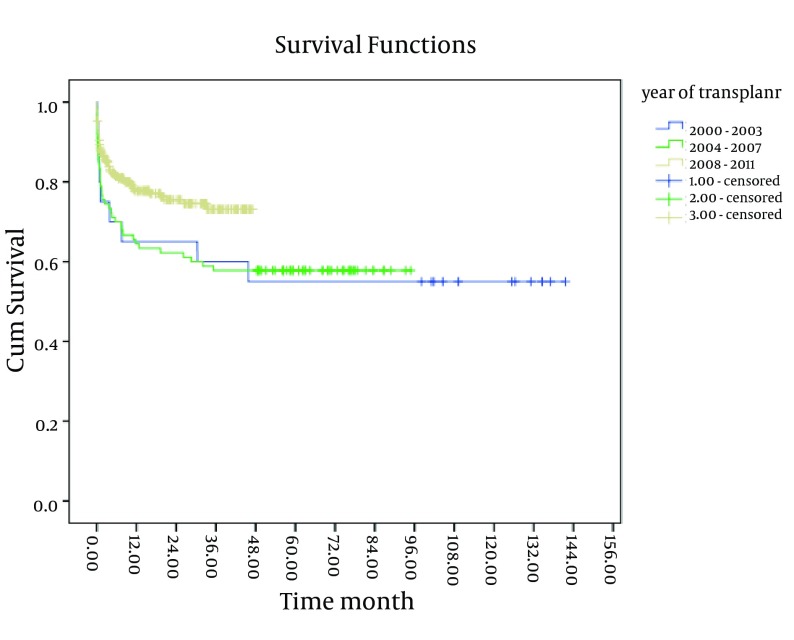
The 10-Year Survival Curve of Patients Below-18-Years-Old Transplanted in the Namazi Hospital Liver Transplant Center Based on the Year of Transplantation

**Figure 6.**
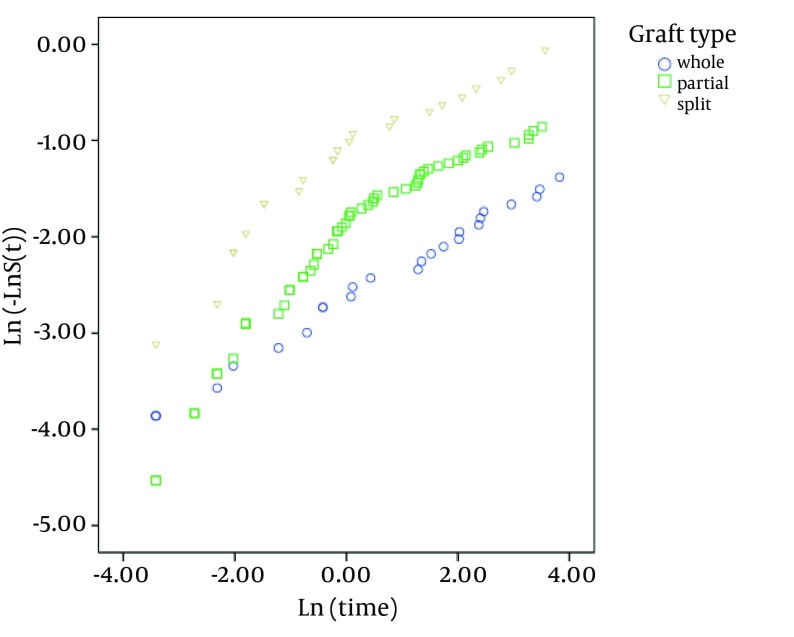
Investigation of the of the Proportional Hazard Assumption Based on the Type of Graft Using the Scatter Plot of [Log (-Log) (t)] on Log (t)

As shown in Figures 3 and 4, existence of post-transplant complications and PELD/MELD scores > 20, decreased the patients’ survival rate (P < 0.05). The patients who were transplanted between 2008 and 2011 had a better survival in comparison to the others (P < 0.05) (Figure 5). In order to assess the proportional hazard assumption, we plotted Log [-logS(t)] on Log (t) for all the variables. However, since all the Figures cannot be presented, the plot of log(-log(t)) on log(t) for the type of graft has been shown in Figure 6. In order to model the effective factors on survival rate, the variables with P < 0.2 and consistent hazard ratios in univariate analysis were entered into the Cox model. These variables included the recipient’s age, the recipient’s weight at transplantation, initial diagnosis of the liver disease, graft type, surgical technique, year of transplantation, and existence of post-transplant complications. Then, Forward Likelihood ratio method was used in order to determine the final model and the results are presented in Table 5. According to the results obtained from the Cox regression model, initial diagnosis of the disease, graft type, PELD/MELD score, and post-transplant complications were effective factors on the survival rate. In comparison with the patients who had received whole transplantation, the hazard ratio of those receiving partial living and split transplantations was 3.36 and 5.1, respectively. The rest of the results are presented in Table 5.

**Table 5.** The Results of the Multivariate Analysis Using the Cox Regression Model

	HR^a^	P value	95%CI for HR	
			**Lower**	**Upper**
**Graft type**				
Whole	1			
Partial	3.36	0.01	1.32	8.5
Split	5.1	0.007	1.57	16.5
**PELD/MELD**				
< 20	1			
≥ 20	2.34	0.02	1.09	5.04
**Complication**				
No	1			
Yes	2.56	0.01	1.2	5.4
**Initial diagnosis**				
Metabolic Disease	1			
Cholestatic	2.1	0.06	0.94	4.9
Hepatitis	0.8	0.7	0.21	3.07
Other	0.34	0.1	0.07	1.6

^a^Hazard ratio

## 5. Discussion

Before 1999, when the first pediatric liver transplantation was performed in Iran, nearly all the children with end-stage liver disease died because of several complications. Through liver transplantation, the children who have no chance for life can live a much longer life. In addition, the progress of surgical techniques and immunosuppressive therapies which caused a revolution in organ transplantation, development of anesthesia care, including less utilization of mechanical ventilation and sedative drugs, led to the improvement of the patients’ survival and reduced their length of ICU stay. In the present study, 1, 5, and 10-year survival of the patients was 73%, 66%, and 66%, respectively, which is lower than the survival rate reported in other studies. For instance, Milglizza conducted a study in Spain and reported the patients’ 1-, 5-, and 10-year survival as 80%, 74%, and 74%, respectively (16). In spite of the fact that we only have a 12-year experience with liver transplantation among this group of patients, the survival rate has increased in our center through the recent years (2008-2011). Considering the fact that 46.3% and 63% of all deaths had occurred in the first and 3 months after the transplantation, respectively, 1-, 5-, and 10-year survival of the patients who had survived 1 month after the transplantation was 84%, 77%, and 77%, respectively, while these measures were obtained as 89%, 81%, and 81%, respectively for those who had survived 3 months after the transplantation. This is comparable with a European study, which reported 1, 5, and 10-year survival of the patients who had survived 3 months after transplantation as 95%, 91%, and 90%, respectively (17). In the current study, among the variables which were assessed, only the recipient’s age group, initial diagnosis of liver disease, recipient’s weight at transplantation, PELD/MELD score, year of transplantation, and existence of post-transplant complications were related to the patients’ survival. In the same line, in the SPLIT study conducted in the U.S. and Canada, initial diagnosis, type of graft, and recipient’s weight were considered as effective factors on the patients’ survival; in a way that the patients who had undergone transplantation due to fulminant hepatic failure and cholestatic diseases as well as those with weight > 2 SD below mean had lower survival rates (18).

Also, in the present study, lower survival rates were related to patients suffering from cholestatic diseases compared to other causes except for malignancies as well as those with weight > 1 SD below mean )P < 0.05.( Moreover, in comparison to other age groups, children below-1-year-old had the lowest survival rate, which is in agreement with other studies, including the one performed by Venick; however, this finding had no significant effect in the Cox model (19, 20). Similar to most previous studies, Wilson’s disease and biliary atresia were the most prevalent indications leading to transplantation in the present study (8, 16). Furthermore, in line with the study conducted by Wallot in Europe, the survival rate of the patients who had undergone liver transplantation due to malignancies was significantly lower than other patients (17). Also, the survival rate was significantly lower in patients receiving split transplants. Other investigations have also revealed the type of graft that is effective on the patients’ survival as well as one of the effective factors on post-transplant survivals in Cox regression model (HR=5.1,p=0.007) (18, 21). The findings of the present study showed no significant relationship between the patients’ survival rate and CHILD categorization. Although this classification is widely used in predicting chronic liver failure (22), it has limitations for predicting the patients’ survival because only a 7-point difference is there between the minimum and maximum disease intensity based on its parameters (23). In this study also, since the patients’ CHILD categories were quite close, it showed no relationship with the patients’ survival. However, a significant relationship was found between the patients’ survival and the PELD/MELD score as well as the existence of post-transplant complications. In the Cox regression model also, a PELD/MELD score > 20 (HR = 2.34, P = 0.02) and existence of post-transplant complications (HR = 2.56, P = 0.001) were effective factors in the survival rate after the transplantation. In this study, the patients’ survival rate did not show a great change 3 years after the transplantation and it remained constant after 5 years because in fact no deaths occurred after 5 years from transplantation. This is consistent with the findings of other studies reporting the highest mortality rate of the patients in the first 90 days after transplantation (24). Therefore, patient care in the first 3 months after transplantation is very important because most of the mortality occurs during this time. The higher mortality rate in the present study might be due to the limited healthcare facilities in our country as well as selection of more critically ill patients in our center. In spite of significant efforts for public education about organ transplantation and donation, due to the shortage of age- and weight-matched deceased donors, living related liver transplantation and split techniques are now more preferably being used for our patients. The most negative survival was due to the poor results of our split liver transplantation during this period. Although living donation has shown a real benefit due to the decreased waiting time, pediatric transplant groups are still faced with technical problems, including a higher incidence of biliary and vascular complications. Survival rate has increased in the recent years (2008-2011) and the number of liver transplantations is increasing in our center. The transplant success rate, which was computed as 1-year survival was obtained to be 73% in the present study, which was quite less than other studies. However, the survival rate of the patients who had survived 3 months after the transplantation was 89% which was comparable with other studies conducted on the issue. Furthermore, cholestatic diseases, split transplant, PELD/MELD score > 20, and existence of post-transplant complications increased the risk of death after the transplantation

## References

[1] Abramson O, Rosenthal P (2000). Current status of pediatric liver transplantation.. Clin Liver Dis..

[2] Busuttil RW, Seu P, Millis JM, Olthoff KM, Hiatt JR, Milewicz A (1991). Liver transplantation in children.. Ann Surg..

[3] Ghobrial RM, Farmer DG, Amersi F, Busuttil RW (2000). Advances in pediatric liver and intestinal transplantation.. Am J Surg..

[4] Muiesan P, Vergani D, Mieli-Vergani G (2007). Liver transplantation in children.. J Hepatol..

[5] Polido WT, Jr, Lee KH, Tay KH, Wong SY, Singh R, Leong SO (2007). Adult living donor liver transplantation in Singapore: the Asian centre for liver diseases and transplantation experience.. Ann Acad Med Singapore..

[6] O'Mahony CA, Goss JA (2012). The future of liver transplantation.. Tex Heart Inst J..

[7] McDiarmid SV (2003). Current status of liver transplantation in children.. Pediatr Clin North Am..

[8] Dehghani SM, Gholami S, Bahador A, Nikeghbalian S, Salahi H, Imanieh MH (2007). Morbidity and mortality of children with chronic liver diseases who were listed for liver transplantation in Iran.. Pediatr Transplant..

[9] Otte JB, de Ville de Goyet J, Reding R, Hausleithner V, Sokal E, Chardot C (1994). Sequential treatment of biliary atresia with Kasai portoenterostomy and liver transplantation: a review.. Hepatology..

[10] Goss JA, Shackleton CR, Swenson K, Satou NL, Nuesse BJ, Imagawa DK (1996). Orthotopic liver transplantation for congenital biliary atresia. An 11-year, single-center experience.. Ann Surg..

[11] McDiarmid SV, Millis MJ, Olthoff KM, So SK (1998). Indications for pediatric liver transplantation.. Pediatr Transplant..

[12] Farmer DG, Venick RS, McDiarmid SV, Ghobrial RM, Gordon SA, Yersiz H (2007). Predictors of outcomes after pediatric liver transplantation: an analysis of more than 800 cases performed at a single institution.. J Am Coll Surg..

[13] Magee JC, Krishnan SM, Benfield MR, Hsu DT, Shneider BL (2008). Pediatric transplantation in the United States, 1997-2006.. Am J Transplant..

[14] Otte JB (2002). History of pediatric liver transplantation. Where are we coming from? Where do we stand?. Pediatr Transplant..

[15] Hoofnagle JH, Lombardero M, Zetterman RK, Lake J, Porayko M, Everhart J (1996). Donor age and outcome of liver transplantation.. Hepatology..

[16] Migliazza L, Lopez Santamaria M, Murcia J, Gamez M, Clavijo J, Camarena C (2000). Long-term survival expectancy after liver transplantation in children.. J Pediatr Surg..

[17] Wallot MA, Mathot M, Janssen M, Holter T, Paul K, Buts JP (2002). Long-term survival and late graft loss in pediatric liver transplant recipients--a 15-year single-center experience.. Liver Transpl..

[18] Martin SR, Atkison P, Anand R, Lindblad AS (2004). Studies of Pediatric Liver Transplantation 2002: patient and graft survival and rejection in pediatric recipients of a first liver transplant in the United States and Canada.. Pediatr Transplant..

[19] Baliga P, Alvarez S, Lindblad A, Zeng L (2004). Posttransplant survival in pediatric fulminant hepatic failure: the SPLIT experience.. Liver Transpl..

[20] Venick RS, Farmer DG, McDiarmid SV, Duffy JP, Gordon SA, Yersiz H (2010). Predictors of survival following liver transplantation in infants: a single-center analysis of more than 200 cases.. Transplantation..

[21] Austin MT, Feurer ID, Chari RS, Gorden DL, Wright JK, Pinson CW (2005). Survival after pediatric liver transplantation: why does living donation offer an advantage?. Arch Surg..

[22] Christensen E, Schlichting P, Fauerholdt L, Gluud C, Andersen PK, Juhl E (1984). Prognostic value of Child-Turcotte criteria in medically treated cirrhosis.. Hepatology..

[23] Kamath PS, Wiesner RH, Malinchoc M, Kremers W, Therneau TM, Kosberg CL (2001). A model to predict survival in patients with end-stage liver disease.. Hepatology..

[24] van der Meulen JH, Lewsey JD, Dawwas MF, Copley LP (2007). Adult orthotopic liver transplantation in the United Kingdom and Ireland between 1994 and 2005.. Transplantation..

